# Predictive Value of the CRP/Albumin Ratio in Mortality Prediction in Hip Fracture Surgery Patients Admitted to Intensive Care

**DOI:** 10.3390/life16081215

**Published:** 2026-07-23

**Authors:** Ayşe Yılmaz, Veysel Garani Soylu, Öztürk Taşkın, Ufuk Demir, Büşra Tanyıldızı Küçük, Özgür Yılmaz, Azra Aytaç, Zahide Doğanay

**Affiliations:** 1 Department of Anestehesiology and Reanimation, Faculty of Medicine, Tinaztepe University, İzmir 35270, Turkey; 2 Department of Intensive Care Medicine, Faculty of Medicine, Kastamonu University, Kastamonu 37150, Turkey; vgsoylu@hotmail.com; 3 Department of Anestehesiology and Reanimation, Faculty of Medicine, Kastamonu University, Kastamonu 37150, Turkey; drozturk275@hotmail.com (Ö.T.); ufukdemir0853@gmail.com (U.D.); btanyldz@gmail.com (B.T.K.); aytacazraa@gmail.com (A.A.); zahidedoganay@yahoo.com (Z.D.); 4 Department of Anestehesiology and Reanimation, Çiğli Training and Research Hospital, İzmir 35620, Türkiye; ege_bey@hotmail.com

**Keywords:** hip fracture, intensive care unit, C-reactive protein/albumin ratio, mortality, prognosis, inflammation

## Abstract

**Background:** Hip fracture surgery patients admitted to the intensive care unit (ICU) have high postoperative mortality, and early risk stratification remains challenging. This study aimed to evaluate the predictive value of the C-reactive protein/albumin ratio (CAR) for 30-day and 90-day mortality in hip fracture surgery patients admitted to the ICU. **Methods:** This retrospective cohort study included 134 adult patients who underwent surgery for hip fracture and were admitted to the ICU between June 2021 and June 2023. Demographic characteristics, comorbidities, APACHE II and SAPS II scores, and laboratory parameters measured within the first 24 h of ICU admission were recorded. CAR was calculated by dividing CRP (mg/L) by albumin (g/dL). The primary outcomes were 30-day and 90-day mortality. Cox proportional hazards regression analysis was performed to identify independent predictors of mortality, and receiver operating characteristic (ROC) curve analysis was used to assess the discriminative ability of CAR. **Results:** The 30-day and 90-day mortality rates were 10.4% and 22.4%, respectively. CAR and serum albumin levels were significantly associated with both 30-day and 90-day mortality (*p* < 0.01), whereas CRP alone was not. In ROC analysis, CAR demonstrated excellent predictive performance for 30-day mortality (AUC: 0.991; 95% CI: 0.975–1.000; cutoff: 38.46; sensitivity: 92.9%; specificity: 92.5%) and good performance for 90-day mortality (AUC: 0.847; 95% CI: 0.776–0.918; cutoff: 25.43). Cox regression analysis identified CAR as an independent predictor of both 30-day (Exp(B) = 1.048, *p* < 0.001) and 90-day mortality (Exp(B) = 1.025, *p* = 0.002). **Conclusions:** The CRP/albumin ratio is a strong and independent predictor of short- and mid-term mortality in hip fracture surgery patients admitted to the ICU. Due to its simplicity and rapid availability, CAR may serve as a practical adjunctive biomarker for early risk stratification in this high-risk population.

## 1. Introduction

Hip fractures are common and serious orthopedic injuries, particularly among elderly individuals, and are associated with significant morbidity and mortality [1]. Age-related frailty, decreased bone density, and an increased risk of falls contribute to the higher incidence of these fractures [2]. Surgical intervention is generally required following a hip fracture; however, many patients are at high risk of multiple systemic complications in both the preoperative and postoperative periods. In particular, patients admitted to the intensive care unit (ICU) may experience postoperative infections, sepsis, organ failures, and nutritional deficiencies, which substantially increase the risk of mortality [3,4,5].

Previous studies have shown the role of biomarkers in prognostic assessment has gained increasing attention. C-reactive protein (CRP) is widely recognized as a marker of systemic inflammation, while albumin provides important information about nutritional status and overall health. The CRP/Albumin ratio (CAR) is considered a combined biomarker reflecting both inflammatory and nutritional status and has emerged as a notable parameter for prognostication in ICU patients [6]. Previous studies have demonstrated the association of CAR with mortality in various clinical conditions, including sepsis, cancer, and cardiovascular diseases.

The aim of this study was to evaluate the predictive value of the CRP/Albumin ratio for 30-day and 90-day mortality in patients undergoing surgery for hip fracture and admitted to the ICU. This study seeks to determine whether CAR can serve as a practical and easily accessible prognostic biomarker to aid clinicians in risk stratification.

## 2. Materials and Methods

### 2.1. Study Design

This study was designed as a retrospective cohort study to investigate the value of the CRP/Albumin ratio in predicting mortality among patients who underwent surgery for hip fracture and were subsequently admitted to the ICU. The study was conducted at the ICU of a tertiary care hospital between June 2021 and June 2023. This study was approved by the Kastamonu University Faculty of Medicine Clinical Research Ethics Committee (approval date: 24 December 2024; approval number: [2024-KAEK-177]). Because the study cohort was assembled retrospectively using de-identified data from existing medical records covering the period June 2021–June 2023, the ethics committee granted a waiver of the requirement for prospective informed consent, in accordance with institutional policy governing retrospective chart-review studies. All procedures were conducted in accordance with the Declaration of Helsinki.

### 2.2. Patient Selection

A total of 134 patients aged ≥18 years who underwent surgical intervention for hip fracture and were admitted to the ICU postoperatively were included in the study. During the study period (June 2021–June 2023), a total of 412 hip fracture surgeries were performed at our institution. Of these patients, 134 (32.5%) were admitted to the ICU postoperatively and constitute our study cohort, indicating that the included population represents a clinically higher-acuity subset. Patients were excluded if they had non-traumatic pathological fractures, active malignancy, chronic inflammatory disease, severe liver failure, nephrotic syndrome, or incomplete laboratory data. Additionally, patients under 18 years of age, those who stayed in the ICU for less than 24 h, or those with incomplete clinical records were also excluded.

### 2.3. Data Collection

Demographic data (age, sex), comorbidities (diabetes mellitus, hypertension, coronary artery disease, and chronic obstructive pulmonary disease), severity scores (APACHE II and SAPS II) recorded at ICU admission, and laboratory parameters measured within the first 24 h of ICU admission were collected retrospectively from patient records.

### 2.4. Laboratory Parameters

C-reactive protein (CRP, mg/L), serum albumin (g/dL), CRP/Albumin ratio (CAR), hemoglobin, hematocrit, and neutrophil-to-lymphocyte ratio (NLR) values were studied. The CRP/Albumin ratio was calculated by dividing the CRP value by the albumin level (mg/L ÷ g/dL).

CRP was measured using a high-sensitivity immunoturbidimetric method (Beckman Coulter AU5800 autoanalyzer Beckman Coulter, Inc., Brea, CA, USA), with a measurement range of 0.1–320 mg/L. Serum albumin was measured using the bromocresol green (BCG) dye-binding method on the same platform. Both parameters were analyzed in the accredited institutional biochemistry laboratory under standard quality control procedures.

This study used CRP in mg/L and albumin in g/dL, reflecting routine laboratory practices at our institution. However, as some studies report CRP in mg/dL, the CAR cutoff values reported here are not directly comparable to those studies (approximately a 10-fold difference). Readers and clinicians should interpret CAR values according to their own institutional units. The adoption of SI units (CRP mg/L, albumin g/L) is recommended to facilitate international standardization.

The Inflammatory Burden Index (IBI) was calculated as the product of leukocyte count and CRP value (mg/L): IBI = Leukocyte × CRP. This composite index aims to assess the systemic inflammatory response by integrating both cellular and acute-phase protein dimensions.

All laboratory parameters were measured within the first 24 h of ICU admission. Patients generally underwent surgery within 24–72 h of hospital admission, and all measurements included in the study were obtained in the postoperative period. Given the effect of surgery on CRP kinetics, this measurement point reflects both the acute inflammatory response and the available nutritional reserve (via albumin). Therefore, postoperative CAR values should be considered an integrated indicator of the systemic response to surgical stress.

### 2.5. Endpoints

The primary endpoints of the study were 30-day and 90-day mortality. Mortality status was verified using hospital records and the national death registry system.

### 2.6. Statistical Analysis

Data were analyzed using an appropriate statistical software package. The distribution of continuous variables was assessed using the Kolmogorov–Smirnov test. Normally distributed data were presented as mean ± standard deviation, while non-normally distributed data were presented as median (minimum–maximum). Categorical variables were expressed as percentages (%). Comparisons between groups were performed using Student’s *t*-test or Mann–Whitney U test for continuous variables and the Chi-square test for categorical variables.

A two-tier variable-selection strategy was applied. For the pre-specified inflammation/nutrition-axis panel (CRP, albumin, CAR, and NLR), an exploratory threshold of *p* < 0.10 in univariate analysis was combined with biological relevance to the study hypothesis. NLR did not individually reach this threshold (*p* = 0.275 for 30-day and *p* = 0.336 for 90-day mortality); however, its two constituent components, neutrophil and lymphocyte counts, were each significantly associated with mortality in univariate analysis (*p* < 0.05). Rather than entering neutrophil count, lymphocyte count, and NLR separately into the same model—which would have introduced substantial collinearity given their mathematical relationship—NLR was pre-specified for inclusion as the single composite index representing this inflammatory relationship, consistent with its established role as an inflammation-based prognostic marker in critically ill and hip fracture populations. For demographic and comorbidity variables screened as potential confounders (age, sex, comorbidities), the conventional threshold of *p* < 0.05 was applied, consistent with the significance level used elsewhere in this study. Variables meeting the respective threshold for their category were CAR, urea, leukocyte count, and hematocrit (*p* < 0.10, inflammation panel); age and sex did not meet the *p* < 0.05 criterion for confounder inclusion. APACHE II and SAPS II scores were not included in the multivariable model, as these composite scores already incorporate several laboratory parameters, including those constituting CAR, and their simultaneous inclusion could introduce multicollinearity; this methodological choice is acknowledged as a limitation of the study.

To identify independent predictors of mortality, Cox proportional hazards regression analysis was conducted, and results were reported as B, Exp(B), and 95% confidence intervals. The predictive performance of the CRP/Albumin ratio for mortality was evaluated using Receiver Operating Characteristic (ROC) curve analysis. Optimal cutoff values were determined by maximizing the Youden Index (J = sensitivity + specificity − 1), with the corresponding area under the curve (AUC), sensitivity, and specificity reported. A *p*-value of <0.05 was considered statistically significant.

## 3. Results

During the study period, 412 hip fracture surgeries were performed at our institution, of which 134 patients (32.5%) were admitted to the ICU postoperatively and were included in the study. The 30-day mortality rate was 10.4% (*n* = 14), and the 90-day mortality rate was 22.4% (*n* = 30). Of these 30 patients, 14 died within the first 30 postoperative days and the remaining 16 died during the 31–90 day interval; Table 1 reports baseline characteristics separately for the 30-day mortality subgroup and for this 31–90 day interval subgroup, rather than for the cumulative 90-day mortality group, in order to characterize how baseline profiles differ across the two mortality groups. Age and sex were not significantly associated with either 30-day or 90-day mortality (*p* > 0.05) (Table 1).

In contrast, the CRP/Albumin ratio (CAR) and serum albumin levels were significantly associated with both 30-day and 90-day mortality (*p* < 0.01). CRP levels alone were not significantly associated with mortality in the univariate analysis (*p* > 0.05). APACHE II scores were significantly higher in patients who died within 30 days (*p* < 0.001) and within 90 days (*p* = 0.002) (Table 2).

In Cox proportional hazards regression analysis, CAR was identified as an independent predictor of both 30-day mortality (B = 0.047, Exp(B) = 1.048, 95% CI: 1.026–1.071, *p* < 0.001) and 90-day mortality (B = 0.025, Exp(B) = 1.025, 95% CI: 1.009–1.042, *p* = 0.002). Leukocyte count was also independently associated with 30-day mortality (B = 0.086, Exp(B) = 1.089, 95% CI: 1.010–1.175, *p* = 0.027) (Table 3 and Table 4). Other variables, including urea, neutrophil-to-lymphocyte ratio, and hematocrit, were not independently associated with mortality.

**Figure 1:**
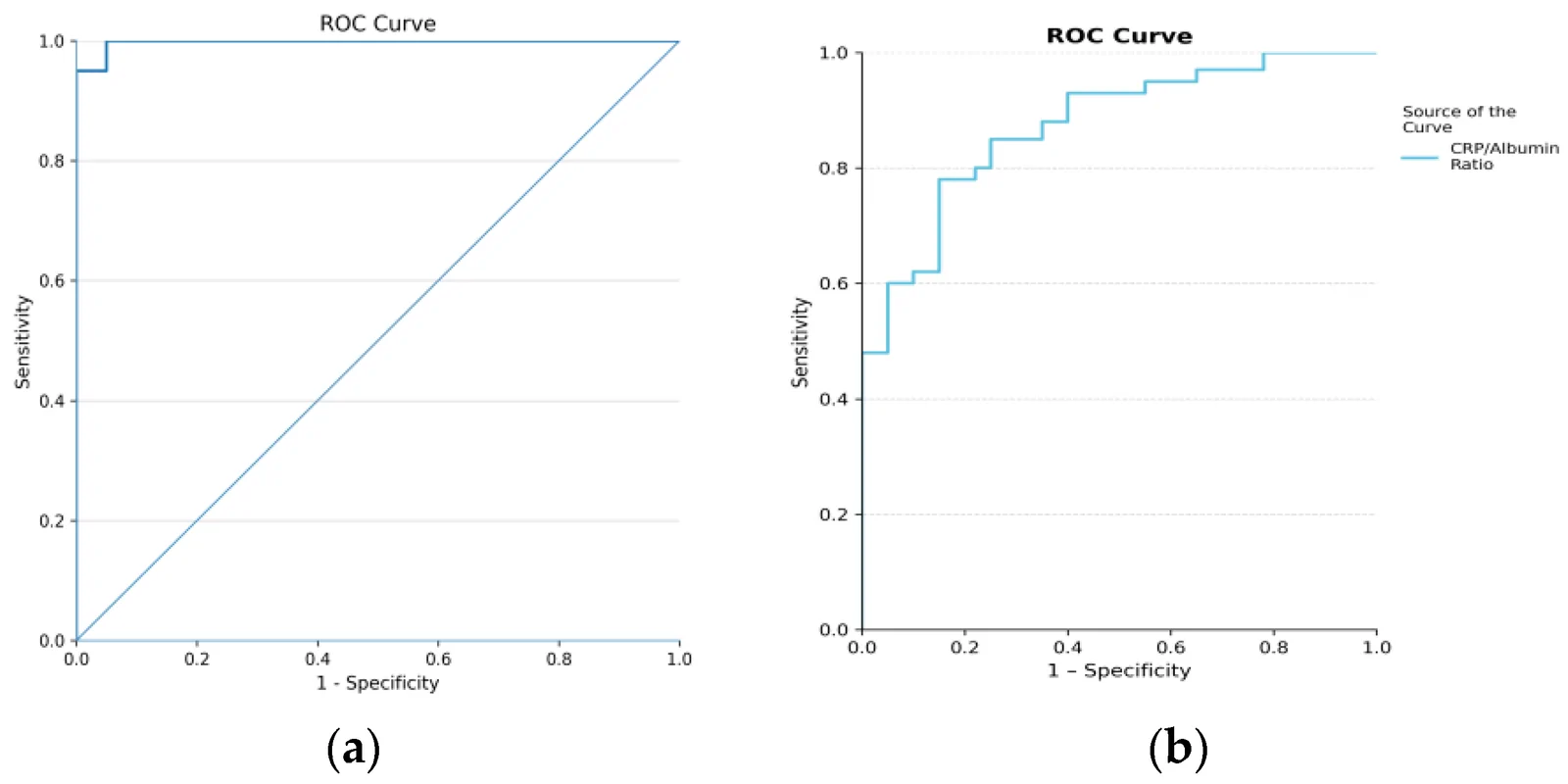
ROC Curve Analysis of the CRP/Albumin Ratio for Predicting 30- and 90-Day Mortality (**a**) 30-Day Mortality; (**b**) 90-Day Mortality.

Receiver operating characteristic (ROC) curve analysis demonstrated that the optimal CAR cutoff value for predicting 30-day mortality was 38.46, yielding an AUC of 0.991 (95% CI: 0.975–1.000), with a sensitivity of 92.9% and a specificity of 92.5%. The optimal cutoff value was determined by maximizing the Youden Index. For 90-day mortality, the optimal CAR cutoff was 25.43, with an AUC of 0.847 (95% CI: 0.776–0.918), sensitivity of 76.7%, and specificity of 76.0% (Figure 1a,b).

## 4. Discussion

This study compared clinical and laboratory parameters between patients who survived and those who died at 30-day and 90-day intervals following hip fracture surgery. Significant differences were observed in APACHE II scores, CRP/Albumin ratio (CAR), and serum albumin levels, highlighting the impact of systemic inflammation and reduced physiological reserve on postoperative mortality.

Hip fractures are associated with high mortality rates, particularly in elderly individuals, with a substantial proportion of deaths attributed to postoperative systemic complications rather than the surgical procedure itself [7]. Patients requiring intensive care admission typically exhibit a more pronounced inflammatory response, which adversely affects outcomes [8]. In this context, the metabolic and inflammatory status at the time of ICU admission becomes a critical determinant of survival [9]. C-reactive protein is a well-established marker of acute-phase inflammation, and previous studies have demonstrated an association between elevated CRP levels and both short- and long-term mortality following hip fracture surgery [10,11]. However, CRP alone may have limited prognostic value. Consistent with this finding, CRP was not independently associated with mortality in the present study. In contrast, *Serum albumin* reflecting nutritional status, inflammatory burden, and overall physiological reserve was significantly associated with mortality, in line with prior studies linking hypoalbuminemia to adverse postoperative outcomes [12,13]. The integration of these two parameters into the CRP/Albumin ratio provides a more comprehensive indicator of systemic inflammation and physiological vulnerability.

Previous studies have identified CAR as a strong predictor of mortality in critically ill populations and in elderly patients undergoing hip fracture surgery [14,15,16,17,18]. Our findings extend this evidence by demonstrating that CAR is independently associated with both 30-day and 90-day mortality. Notably, CAR demonstrated excellent discriminative performance for early mortality. However, this finding should be interpreted with caution due to the limited number of events and the retrospective design of the study.

The stronger association between CAR and early postoperative mortality suggests that acute inflammatory responses may play a more prominent role in short-term outcomes, whereas longer-term mortality may be increasingly influenced by underlying frailty and pre-existing comorbidities [19].

Age was not statistically significantly associated with either 30-day or 90-day mortality in our cohort. While this may appear counterintuitive, it should be noted that the statistical power to detect such an association is limited, particularly for 30-day mortality with only 14 events. Furthermore, it has been proposed that in elderly populations, physiological reserve, inflammatory burden, and nutritional status, all of which are reflected by CAR, may be more determinant of mortality outcomes than chronological age alone.

Although APACHE II is a robust prognostic tool, CAR offers important clinical advantages owing to its simplicity, rapid availability, and ease of calculation. In ICU settings where timely risk stratification is essential, CAR may serve as a valuable adjunct to established severity scoring systems. Early identification of high-risk patients using CAR may facilitate closer monitoring and targeted interventions, including optimized nutritional support and hemodynamic management, potentially improving postoperative outcomes [20]. Consistent with this, a recent large-scale ICU cohort study also demonstrated that CAR independently predicted both 28-day and 1-year mortality and outperformed the SOFA score in predicting long-term outcomes [21].

The strong association between hypoalbuminemia and mortality observed in our study also highlights the potential role of nutritional interventions in this vulnerable population. As the study was retrospective in design, no standardized perioperative nutritional protocol was implemented. However, some studies have shown that high-protein oral supplementation and perioperative carbohydrate loading reduce complications and shorten hospital stay in hip fracture patients. CAR may serve as a practical tool to identify the subgroup of patients most in need of targeted nutritional intervention. Future studies are encouraged to prospectively evaluate the impact of targeted nutritional protocols on mortality in patients with elevated CAR values.

This study has several limitations that should be considered. First, its retrospective design and single-center setting may limit the generalizability of the findings. Additionally, the relatively small sample size reduces the statistical power of the results. Another important limitation is that CRP and albumin levels were measured at ICU admission, which restricts the ability to assess their dynamic changes over time. Due to the limited sample size, bootstrap-based internal validation of the ROC analysis was not performed, which represents a further limitation of this study. The high AUC value (0.991) obtained for 30-day mortality may reflect overfitting and optimism bias associated with the small number of outcome events (*n* = 14). Therefore, the ROC findings for 30-day mortality require external validation in larger cohorts. Additionally, APACHE II and SAPS II were not included in the multivariable model due to the risk of multicollinearity with CAR components; this represents a methodological limitation that should be addressed in future studies. Furthermore, the unit combination used in this study (CRP in mg/L and albumin in g/dL) produces CAR values approximately 10-fold higher than those reported in studies using CRP in mg/dL, which limits direct comparison with the existing literature. Therefore, future multicenter prospective studies with larger patient cohorts are needed to better clarify the role of CAR in clinical decision-making.

## 5. Conclusions

The CRP/Albumin ratio is a strong predictive factor for 30-day and 90-day mortality in hip fracture surgery patients admitted to intensive care. Incorporating CAR into clinical practice may improve early risk stratification and prognostic assessment.

## Figures and Tables

**Table 1:** Baseline Demographic and Clinical Characteristics of Patients by 30-Day and 31–90-Day Mortality Outcomes.

Variable	Total (*N* = 134)	30-Day Mortality (*n* = 14)	*p*	31–90-Day Mortality (*n* = 16)	*p*
Age (years)	75.4 ±16.9	84.3 ±9.9	0.225	69.2 ±14.8	0.073
Gender, Female/Male	68 (50.7%)/66 (49.3%)	6 (42.9%)/8 (57.1%)	–	12 (75%)/4 (25%)	–
Comorbidities, Yes/No	104 (77.6%)/30 (22.4%)	9 (64.3%)/5 (35.7%)	0.206	16 (100%)/0 (0%)	0.393
Diabetes Mellitus, Yes/No	27 (20.1%)/107 (79.9%)	2 (14.3%)/12 (85.7%)	0.563	5 (31.3%)/11 (68.7%)	0.622
Hypertension, Yes/No	53 (39.6%)/81 (60.4%)	4 (28.6%)/10 (71.4%)	0.375	10 (62.5%)/6 (37.5%)	0.366
Malignancy, Yes/No	7 (5.2%)/127 (94.8%)	1 (7.1%)/13 (92.9%)	0.733	2 (12.5%)/14 (87.5%)	0.182
Cerebrovascular Disease, Yes/No	8 (6%)/126 (94%)	0 (0%)/14 (100%)	0.319	4 (25%)/12 (75%)	0.053
Chronic Kidney Disease, Yes/No	5 (3.7%)/129 (96.3%)	0 (0%)/14 (100%)	0.436	1 (6.3%)/15 (93.7%)	0.896
Cardiac Problem, Yes/No	42 (31.3%)/92 (68.7%)	6 (42.8%)/8 (57.2%)	0.326	6 (37.5%)/10 (62.5%)	0.246
Respiratory Disease, Yes/No	20 (14.9%)/114 (85.1%)	0 (0%)/14 (100%)	0.319	3 (18.7%)/13 (81.3%)	0.390

**Table 2:** Relationship of Clinical and Laboratory Parameters with 30-Day and 90-Day Mortality.

Parameter	30-Day Survivors	30-Day Non-Survivors	*p*	90-Day Survivors	90-Day Non-Survivors	*p*
APACHE II	17 (15–20)	21 (19.75–28)	<0.001	17 (15–20)	20 (18.25–25.25)	0.002
SAPS II	35 (28–45)	42 (26–56)	0.008	34 (28–41.75)	43 (29.5–52.5)	0.018
CRP/Albumin	12.54 (4.4–27.32)	98.47 (67.1–107.46)	0.003	14.22 (11.45–22.46)	37.07 (26.16–97.19)	<0.01
CRP (mg/L)	44 (16.37–87.57)	107 (71.25–263.75)	0.087	39.5 (13.95–77.37)	113.5 (79.6–205)	0.255
Albumin (g/dL)	3.3 (3.05–3.7)	2.4 (2.12–3.02)	<0.001	3.3 (3.01–3.8)	3.1 (2.4–3.3)	0.004
BUN (mg/dL)	44 (33–61.75)	61 (30–121)	0.022	41 (32–58.75)	64 (38–104.5)	0.257
Creatinine (mg/dL)	0.91 (0.65–1.24)	0.87 (0.68–1.31)	0.025	0.9 (0.61–1.21)	1.02 (0.74–1.42)	0.099
pH	7.36 (7.32–7.40)	7.35 (7.21–7.43)	<0.001	7.36 (7.32–7.39)	7.37 (7.29–7.42)	0.029
Lactate (mmol/L)	1.6 (1.1–2.4)	2.8 (1.77–4.12)	0.013	1.6 (1.12–2.67)	1.8 (1.25–2.8)	0.056
Neutrophil (/mm^3^)	7995 (5450–9940)	21,850 (17,400–27,770)	0.024	7795 (5300–9800)	14,450 (8180–22,480)	0.181
Lymphocyte (/mm^3^)	930 (690–1390)	410 (360–612)	0.021	915 (630–1390)	755 (410–1040)	0.194
Neutrophil/Lymphocyte Ratio	8.23 (5.27–12.26)	52.99 (38.53–94.46)	0.275	7.68 (5–12.42)	16.75 (8.37–52.47)	0.336
Hospital Stay (days)	6.5 (5–10)	10.5 (4–19.75)	0.020	6 (1–9)	11 (5–22)	0.015
ICU Stay (days)	2 (1–3)	3.5 (2–13)	0.003	4 (2–10)	6 (4–10)	0.001
Inflammatory Burden Index	291.15 (90.09–774.78)	12,083.63 (6236.27–21,684.59)	0.318	261.07 (87.06–708.73)	1541.50 (712.96–11,527.65)	0.318

**Table 3:** Determinants of 30-Day Mortality: Cox Proportional Hazards Model.

Variable	B	SE	Wald	df	*p*	Exp(B)	95% CI for Exp(B)
CRP/Albumin	0.047	0.011	18.331	1	<0.001	1.048	1.026–1.071
Urea	−0.008	0.005	2.826	1	0.093	0.992	0.982–1.001
Neutrophil/Lymphocyte Ratio	−0.499	0.772	0.418	1	0.518	0.607	0.134–2.757
Leukocyte	0.086	0.039	4.891	1	0.027	1.089	1.010–1.175
Hematocrit	−0.095	0.059	2.610	1	0.106	0.909	0.810–1.021

**Table 4:** Determinants of 90-Day Mortality: Cox Proportional Hazards Model.

Variable	B	SE	Wald	df	*p*	Exp(B)	95% CI for Exp(B)
CRP/Albumin	0.025	0.008	9.561	1	0.002	1.025	1.009–1.042
Urea	0.002	0.004	0.386	1	0.535	1.002	0.995–1.009
Neutrophil/Lymphocyte Ratio	0.005	0.015	0.098	1	0.755	1.005	0.975–1.036
Leukocyte	0.015	0.027	0.326	1	0.568	1.015	0.964–1.070
Hematocrit	−0.034	0.034	1.045	1	0.307	0.966	0.905–1.032

## Data Availability

The datasets generated and/or analyzed in this study are not publicly available due to institutional and confidentiality restrictions, but may be obtained from the corresponding author upon reasonable request. All data were obtained from the electronic medical records system of Hospital and contain patient information that cannot be publicly shared in accordance with the Turkish Personal Data Protection Law.
